# Toward a refined genotype–phenotype classification scheme for the international consensus classification of Focal Cortical Dysplasia

**DOI:** 10.1111/bpa.12956

**Published:** 2021-07-01

**Authors:** Ingmar Blumcke, Fernando Cendes, Hajime Miyata, Maria Thom, Eleonora Aronica, Imad Najm

**Affiliations:** ^1^ Department of Neuropathology University Hospital Erlangen Friedrich‐Alexander‐University Erlangen‐Nürnberg Erlangen Germany; ^2^ Epilepsy Center Cleveland Clinic Foundation Cleveland OH USA; ^3^ Department of Neurology University of Campinas—UNICAMP Campinas SP Brazil; ^4^ Department of Neuropathology Research Institute for Brain and Blood Vessels Akita Cerebrospinal and Cardiovascular Center Akita Japan; ^5^ Department of Neuropathology Institute of Neurology, University College London London UK; ^6^ Department of (Neuro)Pathology Amsterdam UMC University of Amsterdam Amsterdam; ^7^ Stichting Epilepsie Instellingen Nederland (SEIN Heemstede The Netherlands

**Keywords:** brain, epilepsy, neuroimaging, neuropathology, seizure

## Abstract

Focal Cortical Dysplasia (FCD) is the most common cause of drug‐resistant focal epilepsy in children and young adults. The diagnosis of currently defined FCD subtypes relies on a histopathological assessment of surgical brain tissue. The many ongoing challenges in the diagnosis of FCD and their various subtypes mandate, however, continuous research and consensus agreement to develop a reliable classification scheme. Advanced neuroimaging and genetic studies have proven to augment the diagnosis of FCD subtypes and should be considered for an integrated clinico‐pathological and molecular classification. In this review, we will discuss the histopathological foundation of the current FCD classification and potential advancements when using genetic analysis of somatic brain mutations in neurosurgically resected brain specimens and postprocessing of presurgical neuroimaging data. Combining clinical, imaging, histopathology, and molecular studies will help to define the disease spectrum better and finally unveil FCD‐specific treatment options.

## Abstract 2

The ILAE classification of Focal Cortical Dysplasia is widely used in clinical practice and research. Diagnostic liability of FCD subtypes remain, however, challenging. Increasing discovery of brain somatic mutations may help to develop a genotype‐phenotype classification scheme for FCD.

## 1 INTRODUCTION

Focal Cortical Dysplasia (FCD) is the most common cause of drug‐resistant focal epilepsy in children and young adults (1). Surgical therapy became a successful option in many patients with FCD (2), in particular, when the lesion is visible on MRI and it co‐localizes with the seizure onset zone. Advanced MRI protocols and postprocessing analyses methods have led to a higher presurgical detection rate of FCD (3). However, any presurgical diagnosis needs a histopathological confirmation by microscopic review of the surgical tissue specimen. With advanced genetic tools and increased sequencing coverage, analysis of surgical brain tissues reveals brain somatic mutations in the mTOR pathway in approximately 60% of FCD (4). These developments will help to reach a next level of integrated genotype–phenotype diagnosis, as it is already routine practice in brain tumors.

**FIGURE 1:**
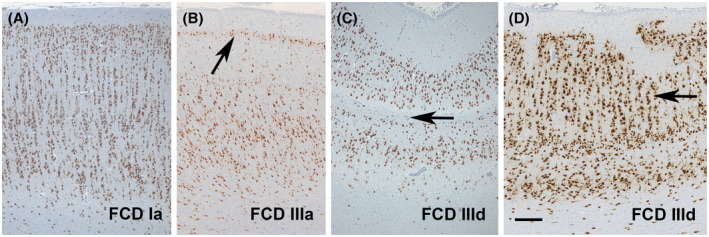
Patterns of architectural dysplasia in FCD subtypes. (A) Note a predominately microcolumnar organization of neurons in layers 3 to 6, that is, FCD ILAE Type 1A, in a 12‐year‐old boy with seizure onset at 2 years of age and difficult‐to‐treat epilepsy. (B) Temporal lobe sclerosis with a prominent presentation of layer 2 caused by loss of pyramidal cells in layer 3 is the characteristic hallmark of FCD 3A and associated with hippocampal sclerosis (62). (C) Loss of layer 4 was observed in a 6‐year‐old boy with remote hypoxemic brain injury. This pattern should be classified as associated FCD 3D (51). (D) Another characteristic pattern of FCD 3D was detected in a patient with perinatal brain injury, demonstrating horizontal and vertical layering abnormalities (arrow). NeuN immunohistochemistry (brown color) with hematoxylin counterstaining. Scale bar in D = 200 µm, applies also to A, B, and C

**FIGURE 2:**
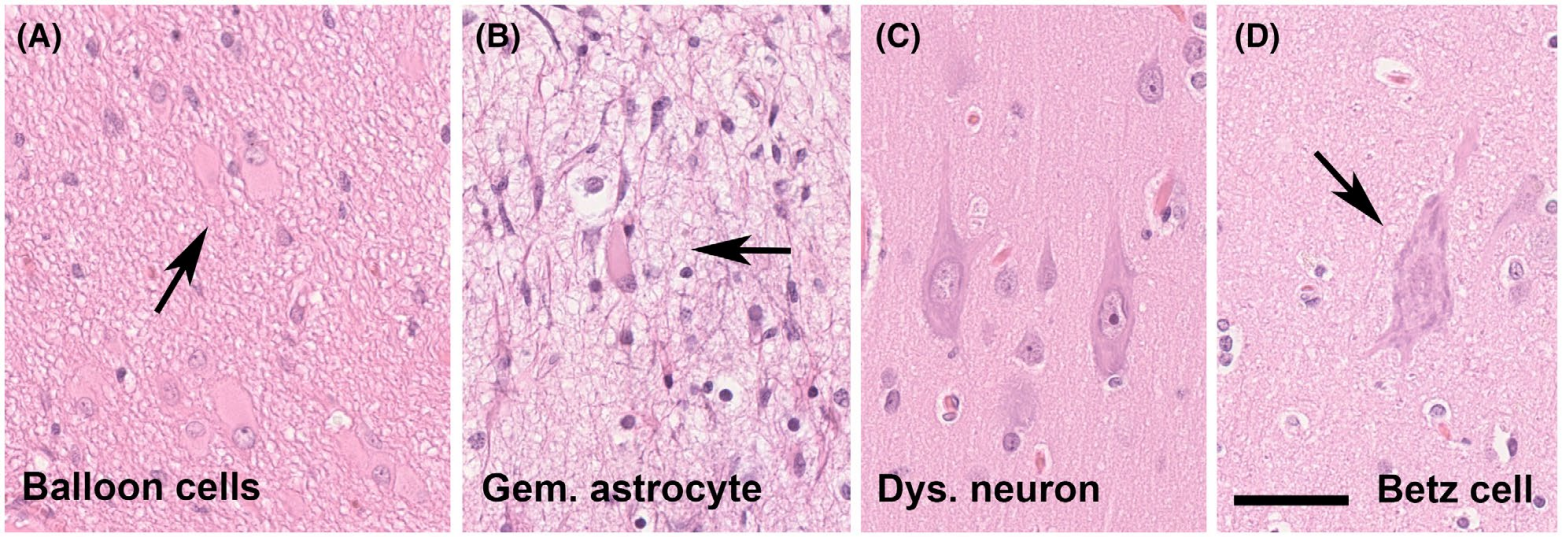
Cytological features of FCD 2 subtypes and its physiologic counterparts. (A) A cluster of balloon cells (arrow) in a 15‐year‐old patient with FCD 2B (H&E staining). (B) A 16‐year‐old patient with focal epilepsy and previous laser ablation. The patient was submitted to a second surgery. Gemistocytic astrocytes were observed in the vicinity of the laser ablation trajectory. (C) Dysmorphic neurons of almost 50 µm diameter seen in a 17‐year‐old patient (same as shown in 2A). (D) This is a giant Betz cell of the primary motor cortex, the Brodmann area 4. These cells resemble dysmorphic neurons. It is imperative, therefore, to know about the surgical resection site and distinguish between occasional norm variants and abundance of abnormally placed and oriented neurons in FCD. Scale bar in D = 50 µm, applies also to A, B, and C

The ILAE consensus classification scheme for FCD specifies three FCD categories based on architectural (e.g., FCD Type 1), cytoarchitectural (e.g., FCD Type 2), and associated principal lesion patterns (e.g., FCD Type 3), and is widely used in clinical practice and research (5). Microscopic hallmarks were currently defined on routine H&E stainings and refer to the aberrant architectural organization of the neocortex (Figure 1). However, many of these neuroanatomical features, including cytological abnormalities of large pyramidal cells, can also be observed in a non‐epileptic human brain (Figure 2). Therefore, it is ever helpful to have independent scientific confirmation of a specific lesion pattern, that is, using molecular studies or animal modeling, to define the disease and further investigate the best therapeutic options.

In daily routine histopathological evaluation of brain tissue obtained from epilepsy surgery, we encounter a large number of difficult‐to‐diagnose specimens and disease patterns scientifically not yet understood. Characteristic examples represent the spectrum of mild malformations of cortical development (mMCD), FCD Type 1, or no lesion cases with gliosis only. New entities are continuously described within this spectrum, such as mild malformation of cortical development with oligodendroglial hyperplasia (MOGHE) (6) and of low‐grade brain tumors (7, 8). However, the clinical significance of such new entities remains to be shown. The increasing revelation of genetic drivers associated with specific lesion patterns is very helpful to advance this field, as it offers new pharmaco‐therapeutic targets. The development of DNA methylation profiling in human brain diseases, in particular brain tumors has been another illuminating methodology to help clarify the clinico‐pathological and molecularly defined disease entities (9, 10).

We propose, therefore, to work toward a reliable genotype–phenotype correlation of well‐characterized FCD categories as they have the propensity for predominant patterns of anatomo‐pathological brain localization, age at disease onset, and molecular signatures. A typical example is that of the recently described bottom‐of‐sulcus FCD Type 2, which is predominantly localized in the frontal lobe (in particular in the superior frontal sulcus). Patients present early with stereotyped hypermotor seizures, and many of these lesions can be associated with mTOR pathway gain‐of‐function mutations (11, 12). Patients with MOGHE, which is predominantly localized also in the frontal lobe, exhibit early seizure onset and the lesion is frequently associated with somatic brain mutations in the SLC35A2 gene (13). In contrast, genetic studies have not yet deciphered a frequent abnormality in FCD 1 subtypes, which also pose major challenges to routine histopathology diagnosis. DNA methylation patterns can distinguish, however, the FCD 1A subtype from most other FCD variants (see Figure 1A; Holthausen et al, unpublished data). We are confident that the catalog of clinico‐pathologically and molecularly defined FCD subtypes will continuously expand, enabling early diagnosis in these difficult patients and lead to the development of targeted treatment options. We will continue this discussion by presenting case vignettes for clinico‐pathologically and molecularly well characterized FCD 2A and FCD 3D subtypes from our own clinical practice.

### 1.1 Case vignette 1: A 50‐year‐old patient with a MRI‐positive FCD 2A and a DEPDC5 germline mutation

**FIGURE 3:**
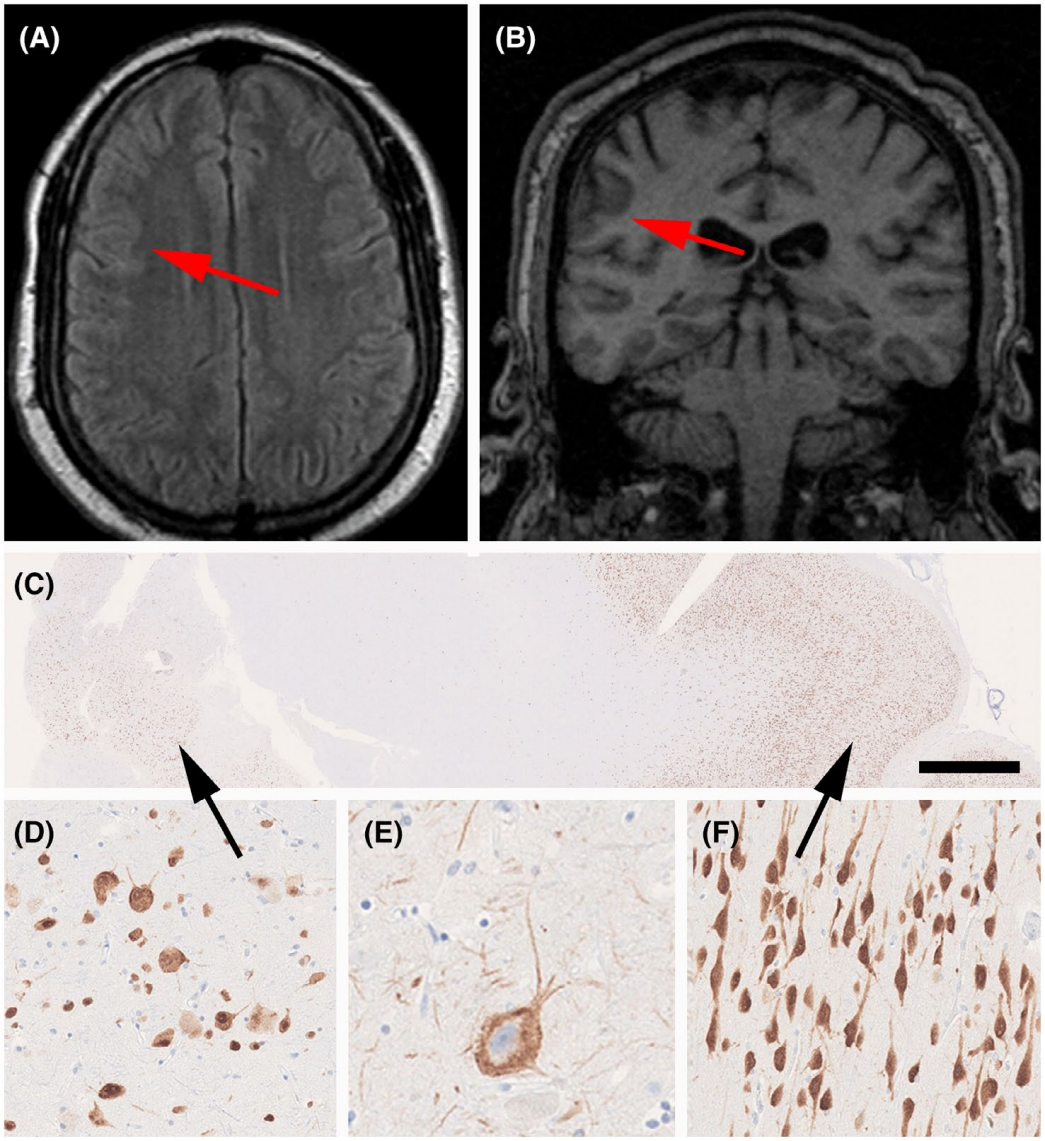
MRI and histopathology findings (see case vignette #1). FLAIR imaging (A) and T1 (B) with red arrows pointing to a subtle lesion with focal cortical thickening and abnormal gyral pattern without hyperintense signal. (C) Low power micrograph of surgical specimen (NeuN immunohistochemistry). The black arrow on the left points to an area of FCD Type 2A, as magnified in (D) and (E) (immunostaining for non‐phosphorylated neurofilament protein). (F) NeuN immunohistochemistry showing well‐oriented pyramidal cells in the adjacent normal‐appearing neocortex (from area highlighted by arrow on the right in 3C). Scale bar in C = 1 mm, in F = 100 µm (applies also to D), in E = 50 µm

We report on a 50‐year‐old male patient with seizure onset at the age of 8 years. He suffered from six to eight focal motor seizures with impaired awareness per month. His son also had seizures. Brain MRI at age 36 was reported as normal. Upon repeated imaging, a subtle area of abnormal gyral pattern, and cortical thickening with no transmantle sign and no hyperintense fluid‐attenuated inversion recovery (FLAIR) signal in the right frontal region was observed (Figure 3A,B). During the presurgical evaluation, this patient had stereo‐EEG recordings. A consensus agreement was reached at the patient management conference to offer a right frontal resection. Histopathological examination of the surgical specimens from the frontal lobe revealed an unlayered neocortex consisting of large and dysmorphic neurons (Figure 3C–E). Immunohistochemical stainings using NeuN (Millipore, Temecula, CA, USA) and non‐phosphorylated neurofilament (BioLegend, San Diego, CA, USA) confirmed the dysplastic nature of cortical pyramidal cells (Figure 3). FCD gene panel testing of MTOR, AKT1, AKT3, PIK3CA, PIK3R2, DEPDC5, TSC1, TSC2, NPRL2, NPRL3, PTEN, BRAF, and SLC35A2 detected a DEPDC5 germline mutation (c.2620C>T, p.Arg874*) in fresh‐frozen brain tissue (from frontal lobe) and paired peripheral blood samples. The patient is seizure‐free 4 years after surgery (Engel class I).

This case highlights a frequent obstacle in clinical practice for the diagnosis of FCD. Several MRIs were performed on this patient and reported negative. This is probably one of the most frequent causes for the exclusion (or at least significant delay) of some patients from surgical consideration. When the patient was finally seen in an epilepsy center, he was 50 years old and suffered from a 42‐year long disease history. Large FCD lesions, particularly those with a clear hyperintense signal on FLAIR images, are easily detected by MRI; however, routine MRIs often miss subtle FCD lesions (14). The frequency of negative MRI in patients with FCD later diagnosed by postoperative histopathology varies considerably depending on the MRI machine used (magnetic field strength and type of head coil), the acquisition protocol, clinical and EEG information, and the experience of the examiner (3, 14, 15, 16). 7T MRI may provide additional information about FCD lesions in patients with or without lesions detected by 1.5 or 3T MRI (15, 17, 18, 19). The increased signal‐to‐noise ratio and susceptibility effects, allowing for better image contrast, higher spatial resolution, and stronger susceptibility contrast, may detect FCD lesions in 16%–32% of previously negative MRI patients (18). Moreover, technical difficulties such as inhomogeneity in 7T images pose challenges for its clinical use (17). In clinical practice, image reconstruction methods and postprocessing from 3D acquisitions with an optimized epilepsy protocol allow better detection of discrete FCD lesions from both 1.5 and 3T MRI (3, 14). Re‐examination of “normal” MRI images in patients with hypometabolism on positron emission tomography with [18F]‐fluorodeoxyglucose (FDG‐PET) in the light of clinical and EEG information may reveal initially undetected subtle FCD lesions (14).

In patients with FCD Type 1, the MRIs are frequently normal or show diffuse or localized cerebral hypoplasia (20). The main MRI features of FCD Type 2, in general, are focal cortical thickening and a mild degree of increased cortical signal intensity in T2‐weighted and FLAIR sequences, blurring of the gray–white matter junction, focal abnormal cortical gyration, and cerebrospinal fluid cleft‐cortical dimple (14, 21, 22). The presence of hyperintense T2‐FLAIR signal in the subcortical white matter with a wedge shape that extends to the ipsilateral ventricle surface (transmantle sign) is a reliable indicator of FCD Type 2B, although not all patients with FCD 2B have a transmantle sign (22, 23, 24). Thus, except for the presence of transmantle sign, the MRI features of FCD 2A and 2B may be indistinguishable. The MRI characteristics in patients with MOGHE are age‐related and seem to change with brain maturation, and include changes in gyral and sulcal morphology, mild cortical thickening, a slight increase in signal intensity in the subcortical white matter, and a mild cortical–subcortical blurring (6, 25, 26, 27). Young children may present with an extensive and pronounced cortical–subcortical blurring that gives a false impression of cortical thickening.(26)

The first DEDPC5 mutation was reported in a series of patients with familial focal epilepsy in 2013 and soon confirmed in other pedigrees (28, 29, 30). It has been confirmed in many other studies and in particular in FCD 2A (31). It has been shown to represent a loss‐of‐function mutation in the GATOR complex DEPDC5 and likely contain a second‐hit brain somatic abnormality, such as a loss of heterozygosity (4, 31). In order to maximize the utility of various studies in patients with pharmacoresistant epilepsy and suspected FCD, we propose that an integration of the various findings in a genotype–phenotype classification (Table 1) will enhance the diagnostic accuracy and help to better stratify patients for research trials and clinical management.

**TABLE 1:** Suggested genotype–phenotype classification of case vignette 1

Neuroimaging findings	A subtle lesion in the right frontal region, characterized by abnormal gyral pattern, cortical thickening, no transmantle sign, and no hyperintense signal
Histopathology	Neocortical dyslamination with dysmorphic neurons, no balloon cells (SMI32 and vimentin immunohistochemistry)
ILAE classification 2011	FCD Type 2A
Molecular‐genetic findings	DEPDC5 germline mutation (c.2620C>T, p.Arg874*) in fresh‐frozen brain tissue and paired peripheral blood samples
Integrated diagnosis	MRI‐positive Focal Cortical Dysplasia 2A, DEPDC5 germline mutation

FCD ILAE Type 2 represents the most common malformations of cortical development (MCD) in surgical series (1) and is associated with favorable surgical outcomes (2). This FCD type was first described by Taylor and colleagues in 1971 (32) as a distinctive isolated malformation in patients with medically intractable epilepsy. Over the past decades, particular attention has been focused on the characteristics of dysmorphic neurons (DN) and balloon cells (BC). Lower overall neuronal densities were observed in the region of dysplasia, and DN displayed variable expression of different cortical layer markers regardless of their laminar location, reflecting their altered migratory pattern, whereas their immunophenotype supports an origin from radial glia and intermediate progenitor cells (33). BC have been shown to express both neuronal and immature glial markers, indicating a failure to differentiate prior to migration into the cortex (34, 35, 36). Several studies using in vitro electrophysiological recording in surgical specimens from patients with FCD Type 2B have shown that BC are essentially electrically silent, whereas neurons display hyper‐excitable intrinsic membrane properties (37, 38). There is also a substantial amount of data demonstrating the persistence of immature features (39), suggesting, in particular, a deregulation of inhibitory synaptic transmission involving altered γ‐aminobutyric acid (GABA)‐A‐mediated currents (40). Interestingly, enrichment of somatic variants of the mTOR pathway has been shown in both DN and BC using advanced laser microdissection or single‐cell DNA sequencing techniques (4, 41).

The pathological features of FCD Type 2 are complex, also involving glial cells, including both oligodendrocytes and astrocytes. White matter pathology with depleted myelin and oligodendroglia represents another major feature of FCD Type 2, indicating impaired oligodendroglial turnover and maturation. Morphological abnormal astroglial cells are often observed in the region of dysplasia, and mounting evidence supports the role of dysfunctional astrocytes (astrogliopathology) in the pathophysiological processes underlying the development of epilepsy. Astrocytes, together with microglial cells highly represented in the FCD Type 2 lesion, also contribute to the induction of major pro‐inflammatory signaling pathways, associated with alterations in the blood–brain barrier and extracellular matrix organization (42, 43, 44). Moreover, FCD Type 2 is also characterized by a prominent activation of adaptive immunity mechanisms involving T‐lymphocytes (43, 45, 46).

Thus, the rapidly expanding knowledge of the complex cellular and molecular features of FCD Type 2 and their mechanistic link to somatic mutations in mTOR pathway regulatory genes underlies the need to improve the diagnostic workup of this FCD type, requiring a multilayer comprehensive diagnostic approach that combines clinical, radiological, histological, and molecular‐genetic characteristics. However, the variable mutation level within the lesion (with often low‐level mosaic mutations) is one of the challenges that we are currently facing in the effort to apply the concept of “integrated diagnosis” to FCD Type 2. Highly standardized tissue processing using representative brain tissue enriched in DN and BC‐derived DNA is required for the genetic diagnosis of FCD Type 2 (4, 47).

### 1.2 Case vignette 2: A 17‐year‐old patient with an MRI‐positive FCD 3D, FCD gene panel negative

**FIGURE 4:**
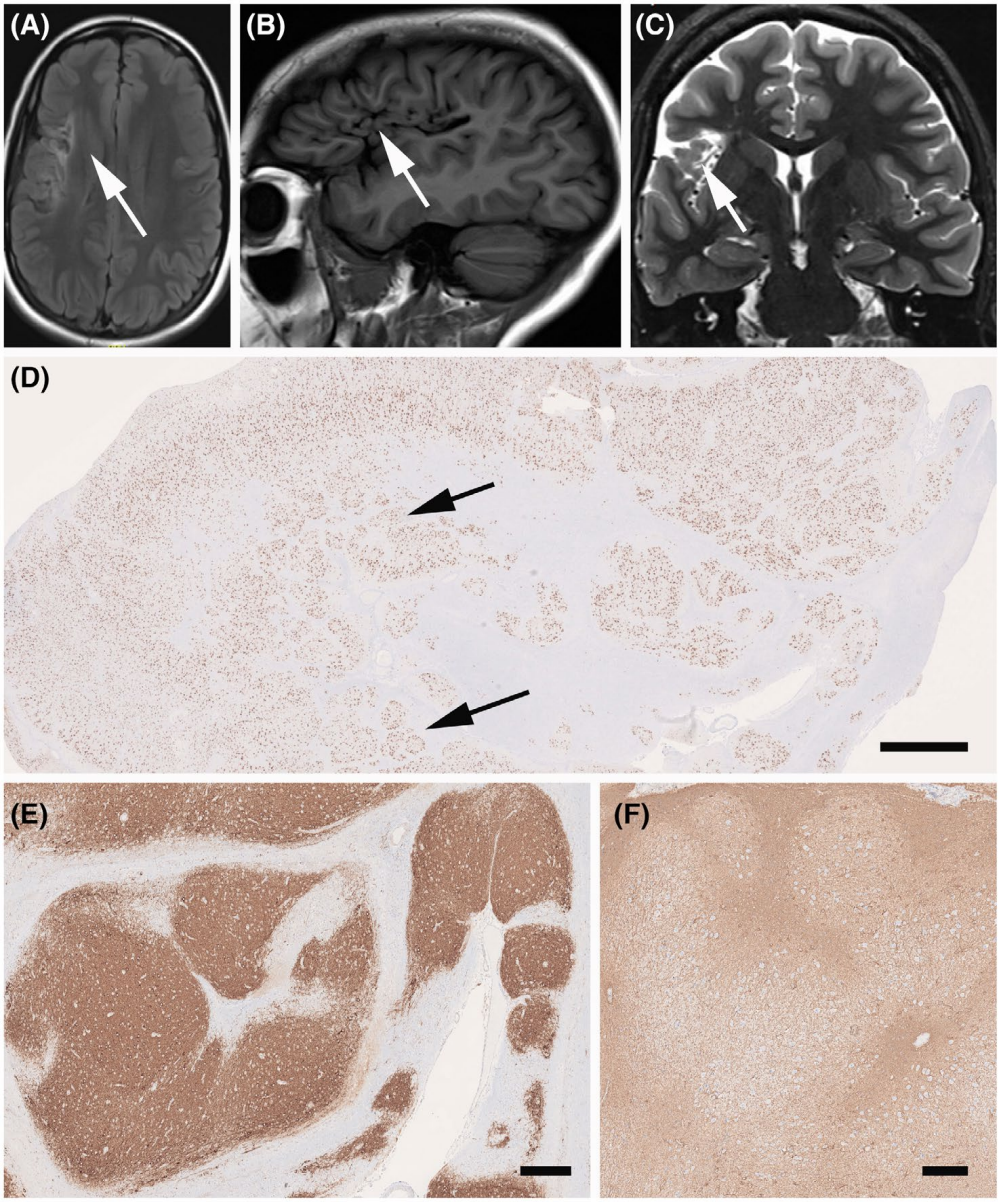
MRI and histopathology findings (see case vignette #2). FLAIR imaging (A), T1 (B), and T2 (C) with encephalomalacia in the right fronto‐parietal operculum and subinsular region, consistent with remote infarction in the right MCA territory (arrows); (D) Low power micrograph of surgical specimen with characteristic neuronal clusters of the cortical architecture (arrows; NeuN immunohistochemistry). MAP2 (E) and GFAP (F) immunohistochemistry revealed the same nodular appearance of the neocortex in FCD Type 3D. Scale bar in D = 1 mm, in E = 100 µm (applies also to D)

We report on a 17‐year‐old female patient with seizure onset at the age of 5 years. She was born at the 35th week of gestation with an ischemic infarct of the right middle cerebral artery (Figure 4). She suffered from face somatosensory auras followed by tonic face seizures and bilateral eyelid flutter or evolving into a generalized tonic–clonic seizure. MRI studies confirmed encephalomalacia in the right frontoparietal operculum and subinsular region, consistent with remote infarction in the territory of the right middle cerebral artery (MCA; Figure 4A–C). During presurgical evaluation, this patient also had stereo‐EEG recordings and intraoperative electrocorticography. A consensus agreement was reached at the patient management conference to offer a right frontal lobe resection. We received the surgical specimen for microscopic review and observed a microscopically detectable lesion in the frontal neocortex showing clusters of neurons and neuropil in the superficial neocortex surrounded by a dense glial scarring. There were no DN or BC. Immunohistochemical stainings using NeuN (Figure 4D), MAP2 (Figure 4E; antibodies provided by Dr. Riederer, Lausanne, Switzerland), and glial fibrillary acidic protein (GFAP; Figure 4F; Dako, Glostrup, Denmark) confirmed an acquired FCD Type 3D subtype. FCD gene panel testing of MTOR, AKT1, AKT3, PIK3CA, PIK3R2, DEPDC5, TSC1, TSC2, NPRL2, NPRL3, PTEN, BRAF, and SLC35A2 detected no mutation in fresh‐frozen brain tissue (from frontal lobe) and paired peripheral blood samples. The patient is seizure‐free with occasional auras since 1 year after surgery (Engel class Ib).

This case highlights the concept of acquired FCD, that is, FCD Type 3 of the ILAE classification system (5). It is generally believed that these acquired lesions do not carry genetic driving mutations, which was also proven in this case. This does not exclude, however, that there is no genetic risk factor present, but this will need a different approach for testing, that is, genome‐wide copy number variations (48). SEEG recordings confirmed the onset of seizures at the lesional site, and we continue to hypothesize that FCD 3D is the underlying structural lesion in need of surgical resection treatment. However, FCD 3D is a difficult‐to‐treat situation as the lesion may extend the MRI visible area, and long‐term disease history decreases the chance of postsurgical seizure control (2). In other cases, FCD 3 subtypes are more debatable, such as FCD 3A associated with hippocampal sclerosis (49) and FCD 3B associated with a brain tumor (7).

Based on the findings of this case and our current understanding to improve clinical diagnosis using a multi‐tiered approach, we would suggest the following classification scheme (Table 2):

**TABLE 2:** Suggested genotype–phenotype classification of case vignette 2

Neuroimaging findings	Encephalomalacia in the right frontoparietal operculum and subinsular region, consistent with remote infarction in the right MCA territory
Histopathology	Glial scarring and cortical dyslamination with neuronal clustering, no dysmorphic neurons, and no balloon cells (SMI32‐ and vimentin immunohistochemistry)
ILAE classification 2011	FCD Type 3D
Molecular‐genetic findings	FCD gene panel negative for MTOR, AKT1, AKT3, PIK3CA, PIK3R2, DEPDC5, TSC1, TSC2, NPRL2, NPRL3, PTEN, BRAF, and SLC35A2 (fresh‐frozen brain tissue and paired peripheral blood samples)
Integrated diagnosis	MRI‐positive Focal Cortical Dysplasia 3D, FCD gene panel negative

FCD Type 3 was introduced into the 2011 ILAE classification to distinguish architectural abnormalities of the neocortex adjacent to an epileptogenic principal lesion from isolated FCD Type 1 (5). A major concern at that time was that such associated FCD Type 3 lesions may result from the same pathomechanism underlying the principle lesion and would not exist otherwise as *bona fide* cortical dysplasia. However, four subtypes were proposed based on the fact that previous publications addressing this topic specifically focused on patients having neocortical architectural abnormalities associated with hippocampal sclerosis (FCD Type 3A), low‐grade developmental brain tumors (FCD Type 3B), vascular malformations (FCD Type 3C), other lesions acquired in early life, that is, encephalitis or perinatal brain insults (FCD Type 3D). FCD Type 3D is a concept adopted from “progressive cortical dysplasia” in young children with shaken infant syndrome (50). Indeed, perinatal injuries are frequent and compromise cortical maturation, known to continue into adolescence. Classic examples are glial scars and abnormal cortical layering with horizontal and radial orientation around porencephalic cysts (Figure 4). This pattern contrasts any traumatic brain injuries occurring at later ages and which do not change the architectural matrix of the neocortex. A distinct clinico‐pathological variant of FCD Type 3D has recently been described in 12 children with remote hypoxic–ischemic injury, histologically showing vertical dyslamination characterized by loss of layer 4 in the occipital neocortex (51), similar to the picture shown in the 2011 ILAE classification as one example of FCD Type 1B. The case published in 2011 was re‐reviewed and re‐assigned to FCD Type 3D (Figure 1C).

All patterns of architectural abnormalities occurring either as FCD ILAE Type 1 or Type 3 can be observed in other disease conditions. As a matter of fact, they even reflect normal architectural components of the human brain, as historically highlighted by the various cortical parcellation schemes (52). Other troublesome differential diagnoses of architectural abnormality include tissue artifacts as a result of oblique tissue cuts as demonstrated by irregular vascular profiles, surgical manipulation including toothpaste phenomena, and bleedings, as well as previous invasive recordings using depth electrodes, subdural grids, or intraoperative electrocorticography. The difficulty of describing a cortical specimen as normal does not exclude any compromised molecular signaling pathway as these may manifest only at the molecular level, that is, DNA methylation signature, and cannot be detected microscopically.

#### 1.2.1 FCD Type 1: Neuropathology and current understanding of the disease

In the setting of drug‐resistant focal epilepsy, architectural abnormalities of the human cerebral neocortex without cellular or “cytoarchitectural” features of DN and BC were first described in 2002 (53). The histological feature seemed distinct from cortical dysplasia of Taylor‐type (32) and was subsequently introduced into the Palmini classification scheme as FCD Type 1 (54). Since then, FCD Type 1 became an increasingly recognized clinical diagnosis, often despite the absence of a readily visible lesion on MRI. Another ongoing debate was poor histopathology agreement for this category (55). Particular areas of conflict posed those patients with an MRI‐visible non‐FCD lesion, that is, hippocampal sclerosis (HS), vascular malformations, and brain tumors. Noteworthy, clinical presentation of FCD Type 1 in patients with early‐onset epilepsy and high seizure frequency is different from that of patients with HS and febrile seizures followed by a latency period, after which seizures may occur only weekly or monthly (56, 57). In addition, neither clinical presentation nor postsurgical outcome was different in patients with HS with or without associated FCD Type 1 (57, 58). Based on these considerations, a previous ILAE Task Force decided to split the large group of FCD Palmini Type I into two newly defined categories (5), that is, FCD ILAE Types 1 and 3. According to the 2011 ILAE classification of FCD, FCD Type 1 remained a specific category showing architectural disorganization of the neocortex likely resulting from compromised neurodevelopment, without evidence of any other epileptogenic principal lesion in the brain examined with either MRI or histopathology. The concept of such FCD Type 1 has been supported by the description of a series of young patients with a severe and difficult‐to‐treat epilepsy phenotype (20, 56). The histopathology signatures of these children share posterior quadrant epilepsy with frequent seizures, disease onset in early life, and histopathologically described abundance of so‐called “microcolumns,” hitherto classified as FCD Type 1A (Figure 1A). Similar patterns of microcolumnar organization of the neocortex were also described in children with genetic defects or inborn metabolic diseases, although with more widespread distribution (59). It was discussed by the Task Force that such microcolumnar organization resembles neuronal radial migration streams during corticogenesis (60, 61) and may result, therefore, from delayed or arrested maturation at mid‐gestation. However, experimental evidence has not yet been provided.

Taking these neurodevelopmental principles in the neocortical organization into consideration, the Task Force then also acknowledged the possibility of compromised horizontal layer organization, which has been assigned as FCD Type 1B (5). Up to date, however, no study conclusively described a clinical phenotype or syndrome for patients with FCD Type 1B. The illustrated case shown in Figure 2 of the 2011 classification was re‐reviewed meanwhile and re‐assigned to FCD Type 3D (Figure 1D) with loss of layer 4 in the occipital lobe associated with perinatal hypoxic injury (51). Another FCD Type 1 subcategory was also established in the 2011 classification scheme, assuming a mixed phenotype of radial and horizontal cortical disorganization, that is, FCD Type 1C. Such a mixed phenotype has been indeed recognized by routine pathology in the neocortex compromised by early perinatal lesions, such as large vascular malformations. Nonetheless, the categories of FCD Type 1B and 1C are often used nowadays, although we have not yet learned about a specific clinical presentation of these patients. They usually remain MRI negative (24) and have not been reported or published before the 2011 ILAE classification of FCD was released. The true existence and potential pathogenic cause will require, therefore, continuous research efforts.

## AUTHOR CONTRIBUTIONS

The authors contribution to the manuscript were as follows: Ingmar Blumcke wrote the introduction, Imad Najm drafted the case vignettes, Fernando Cendes drafted the MRI sections, Maria Thom drafted the section on FCD 1, Eleonora Aronica on FCD 2 and Hajime Miyata on FCD 3. Ingmar Blumcke has finalized the manuscript. All authors reviewed, edited, and approved.

## Data Availability

Data sharing is not applicable to this article as no datasets were generated or analyzed during the current study.
